# Sensitizing the Efficiency of ICIs by Neoantigen mRNA Vaccines for HCC Treatment

**DOI:** 10.3390/pharmaceutics16010059

**Published:** 2023-12-29

**Authors:** Rui Han, Yuqian Wang, Lingeng Lu

**Affiliations:** 1 Department of Chinese Medicine Oncology, The First Affiliated Hospital of Naval Medical University, Shanghai 200433, China; 2 Department of Chinese Medicine, Naval Medical University, Shanghai 200433, China; 3 Department of Oncology, The First Hospital Affiliated to Guangzhou University of Chinese Medicine, Guangzhou 510405, China; 4 Department of Chronic Disease Epidemiology, Yale School of Public Health, Yale University, New Haven, CT 06520-8034, USA; 5 School of Medicine, Center for Biomedical Data Science, New Haven, CT 06520-8034, USA; 6 Yale Cancer Center, Yale University, New Haven, CT 06520-8034, USA

**Keywords:** neoantigen mRNA vaccines, immune checkpoint inhibitor, HCC, combined therapy, novel therapeutic strategy

## Abstract

This study builds upon the groundbreaking mRNA vaccine Nobel Prize win in 2023 for COVID-19 prevention, paving the way for next-generation mRNA cancer vaccines to revolutionize immunotherapy. Despite the existing challenges, such as the presence of a suppressive tumor microenvironment and the identification of cancer-associated antigens, recent results from the KEYNOTE-942 trial have successfully demonstrated the effectiveness of mRNA-based cancer treatments, providing clinical evidence for the first time. This trial aimed to evaluate the efficacy and safety of combining immune checkpoint inhibitors with mRNA-based therapies in treating cancer. This advancement undeniably represents new hope for hepatocellular carcinoma (HCC) patients. However, progress in this field remains limited. In this article, we summarized the current state of applying immune checkpoint inhibitors (ICIs) combined with neoantigen mRNA vaccines. Additionally, we discussed potential targets for designing novel mRNA vaccines and potential mRNA vaccine delivery vehicles. The objective of this article is to inspire enthusiasm for the exploration of innovative therapeutic strategies that combine ICIs with neoantigen mRNA vaccines for HCC treatment and HCC prevention.

## 1. Current Role of Neoantigen mRNA Vaccine in HCC Treatment

The groundbreaking use of mRNA vaccines in preventing COVID-19 was awarded the 2023 Nobel Prize [1]. This achievement has also heightened the urgency to apply gene therapy in tumor treatments, as it has demonstrated remarkable regulatory capabilities on the immune system [2]. Moreover, a neoantigen mRNA vaccine is a personalized cancer vaccine that aims to target neoantigens. Neoantigens are unique antigens found on the surface of cancer cells due to somatic mutations [3,4]. These genetic alterations are unique to each patient’s cancer, and the neoantigen mRNA vaccine is formulated by analyzing the patient’s tumor genome to pinpoint the mutations responsible for generating the neoantigens. The vaccine is then designed to encode these neoantigens, which are produced by the mutations, and delivered to the patient’s immune system via mRNA [5]. The objective of the neoantigen mRNA vaccine is to provoke a precise immune reaction against the cancer cells while minimizing harm to healthy cells. This method has been considered to be a promising strategy for personalized cancer treatment [6].

Numerous individuals are diagnosed with advanced HCC at the point of diagnosis because its early stages lack distinct symptoms. Even the novel therapeutic strategy of Pembrolizumab (PD-1 inhibitor) plus Lenvatinib is not effective enough for patients with advanced HCC [7]. With the evolution of tumor immunology methods and the strides made in molecular biology, as exemplified by mRNA vaccines, tumor immunotherapy provides a renewed sense of optimism for patients. mRNA-based cancer vaccines have been employed to combat a diverse array of cancer types and have been designed to carry various target proteins [8]. These proteins include those aimed at tumor-associated antigens (TAA), tumor-specific antigens (TSA), and immunostimulatory molecules. As bioinformatics for second-generation sequencing continues to advance and precision medicine becomes increasingly prominent in the field of oncology, there is a growing focus on the creation of personalized mRNA cancer vaccines that express tumor-specific antigens (TSAs) [9].

Here, we focus on the latest advancements in combining ICIs with neoantigen mRNA vaccines for HCC treatment. We examine new possibilities for mRNA vaccine development and their delivery methods. The article aims to enhance interest in novel therapeutic strategies, particularly the combined use of ICIs and neoantigen mRNA vaccines for HCC treatment and prevention, inspired by the promising results of the KEYNOTE-942 trial [10,11]. The conclusion emphasizes the potential of mRNA vaccines to improve treatment efficacy and overcome current challenges in cancer immunotherapy, while acknowledging the need for efficient production, specialized storage, and regulatory compliance. The paper also calls for continued research and industry collaboration to fully realize the potential of mRNA vaccines in liver cancer treatment.

Here, we focus on summarizing the current state of applying ICIs with these vaccines, discussing potential targets for designing novel mRNA vaccines, and evaluating potential delivery vehicles for mRNA vaccines. The paper aims to inspire innovative therapeutic strategies for HCC treatment and prevention, building upon the promising results of the KEYNOTE-942 trial.

## 2. Revelation on HCC Treatment Given by KEYNOTE-942 Trial

A noteworthy achievement has been recently attained through the utilization of neoantigen mRNA vaccines, which represent an innovative strategy to amplify the effectiveness of immune checkpoint inhibitors (ICIs) in addressing solid cancer cases, as demonstrated in the KEYNOTE-942 clinical trial [12]. In detail, this phase II clinical trial, also known as the mRNA-4157-P201 trial, assessed the safety and effectiveness of a personalized cancer vaccine called mRNA-4157 when used alongside pembrolizumab, a specific class of immune checkpoint inhibitors (PD-1 inhibitors), as a therapeutic approach for patients diagnosed with head and neck squamous cell carcinoma (HNSCC). This trial encompassed individuals diagnosed with either locally advanced or metastatic HNSCC who had not undergone any prior systemic treatment for their condition. Patients received the mRNA-4157 vaccine, engineered to trigger the immune system’s recognition and targeting of tumor cells unique to each patient, along with pembrolizumab, which essentially unleashes the immune system’s potential by removing the constraints that impede its ability to combat cancer cells efficiently. The trial showed promising results, demonstrating a disease control rate (DCR) of 67% and an overall response rate (ORR) of 33%. Moreover, the combination therapy appeared to be well tolerated, with no new safety signals identified [13].

The KEYNOTE-942 trial has preliminarily and successfully demonstrated the potential of personalized cancer vaccines when used alongside ICIs such as pembrolizumab in enhancing the prospects for patients dealing with HNSCC, a difficult-to-treat cancer with few effective treatment options. The mRNA-4157 vaccine is particularly promising because it is designed to be tailored to each patient’s specific tumor mutations, potentially increasing its effectiveness, which opens the gate for applying such a novel therapeutic combination in various other cancer types, including hepatocellular carcinoma (HCC), a cancer typically resistant to conventional therapies. The trial’s focus on tailored vaccines and immune checkpoint inhibitors like pembrolizumab could lead to more effective, personalized treatment strategies, potentially transforming the therapeutic landscape for HCC patients.

HCC, being another challenging-to-treat cancer and ranking as the fourth leading cause of cancer-related mortality worldwide, is anticipated to continue increasing in incidence rates, especially in countries such as the United States and China, until the year 2030 [14]. Based on the evidence, ICI-based combination therapies have shown promise in the treatment of HCC, and have become a main research direction of this field. For instance, one approach involves combining a PD-1 inhibitor with a CTLA-4 inhibitor, which has shown improved response rates and survival outcomes in clinical trials when compared to single-agent therapies [15]. Other potential combination therapies for HCC include combining PD-1 inhibitors with tyrosine kinase inhibitors, vascular endothelial growth factor (VEGF) inhibitors, carbonic anhydrase XII (CAXII) inhibitor, histone deacetylase (HDAC) inhibitor, or radiation therapy [16]. However, ongoing research is still focused on identifying the most effective combinations. Based on the findings of the KEYNOTE-942 trial new findings have been revealed, that more attention should be given to the research of treating HCC using neoantigen mRNA vaccines plus ICIs.

## 3. Conception of Combing Neoantigen mRNA Vaccine and ICIs for Treating HCC

**Figure 1:**
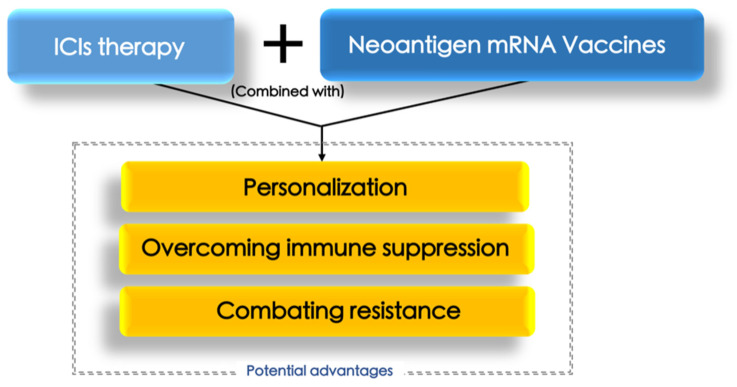
The potential advantages of adding neoantigen mRNA vaccine in ICI-based therapy for treating HCC, which are overcoming immune suppression, personalization, and combating resistance.

From the perspective of mechanism, neoantigens are protein fragments that appear on the surface of cancer cells due to unique mutations within the tumor [17]. These mutations are specific to each patient, rendering neoantigens an ideal target for personalized cancer vaccines. Neoantigen mRNA vaccines operate by introducing synthetic mRNA encoding these patient-specific neoantigens into the body [17]. Antigen-presenting cells (APCs) take up this mRNA and translate it into neoantigen proteins. These proteins are subsequently presented on the surface of APCs, initiating an immune response [18]. In essence, the immune system identifies these neoantigens as foreign or abnormal, leading to the activation of T cells. T cells, specialized immune cells, then identify and eliminate cancer cells displaying these neoantigens [18]. Moreover, neoantigen vaccines are highly personalized, constructed based on the distinct mutations discovered within a patient’s tumor. This approach minimizes the risk of unintended effects while maximizing the immune response against the specific cancer cells [17,18]. Regarding ICI therapy, it functions by obstructing interactions between immune checkpoint proteins, such as PD-1 (programmed cell death protein 1) and CTLA-4 (cytotoxic T-lymphocyte-associated antigen 4), and their corresponding receptors on T cells [19]. This inhibition effectively releases the “brakes” on T cells, enabling them to recognize and attack cancer cells [19]. In vivo evidence has proved that, in HCC cells, heterogeneity exists among the CD4+ T cells infiltrating tumors and tumor-draining lymph nodes, and a particular subset has a defined gene signature that was enriched in patients with intrinsic resistance to ICIs [20]. However, those immunostimulatory and inhibitory epitopes can be identified through high-throughput ex vivo screening assays involving autologous T cells and APCs. Therefore, developing a certain vaccine for properly inducing CD4+ T cell phenotypes has been considered to act as a sensitizer for ICI therapy [21,22]. The combination of neoantigen mRNA vaccines and ICI therapy holds significant promise for several reasons (Figure 1):

### 3.1. Overcoming Immune Suppression

Some tumors create an immunosuppressive microenvironment that compromises the efficacy of ICIs when used alone. Neoantigen vaccines can serve to “prime” the immune system within the tumor, rendering it more receptive to ICI therapy. As an immunosuppressive tumor, HCC often possesses an exhausted phenotype of TILs with low cytotoxic activity [23,24]. However, as one of the most commonly mutated genes in HCC, TP53 has been considered as a potential target for neoantigen vaccines. Evidence has shown that patients with a TP53 neoantigen have longer overall survival, higher immune score, better prognosis, higher cytotoxic lymphocyte (CTL) infiltration, and a higher cytolytic activity score than patients without [25]. Moreover, in a recent study, researchers constructed a Db/Sp244-252/R251H neoantigen epitope (changing an amino acid site) in the EndoB2-Sp protein and found that a single injection of the EndoB2-Sp expression vector into C57Bl/6j mice could effectively induce activation of IFN-γ + CD8+ T cells specifically targeting the neoantigen epitope (Db/Sp244-252/R251H). The expression of Db/Sp244-252/R251H within the core antigen of assembly-deficient HBV induced a considerable number of CD8+ T cells specifically targeting Db/Sp244-252/R251H compared with the EndoB2-Sp vaccine [26].

### 3.2. Personalization

Neoantigen vaccines are tailored to the unique characteristics of each patient’s tumor, enhancing their specificity. When used in conjunction with ICIs, these vaccines broaden the activation of the immune system, targeting not only neoantigens but also other potential tumor-associated antigens. Furthermore, a recent clinical study provided 10 HCC patients at high risk of postoperative recurrence with personalized neoantigen vaccines [27]. Eventually, this study found that no obvious adverse events were observed during neoantigen vaccinations. Additionally, among seven of the patients who received all of the planned neoantigen vaccinations, five of them demonstrated neoantigen-induced T cell responses and had significantly longer recurrence-free survival (RFS) after radical surgery than the other five patients without responsive neoantigens or only with prime vaccination and propensity scores matching the control patients (*p* = 0.035). Thus, the safety and efficiency of those individualized vaccines has been preliminarily displayed in suppressing the recurrence of HCC [27]. In the context of melanoma, a vaccine targeting personalized neoantigens has been tested [28]. The vaccine was developed by conducting whole-exome sequencing of DNA from matched normal and tumor cells from individual patients to identify somatic mutations. These mutations were further validated by RNA-seq in the tumor. Subsequently, a vaccine targeting up to 20 predicted personal tumor neoantigens was generated. Among six vaccinated patients, four remained recurrence-free 25 months after vaccination. In cases where recurrence occurred, two patients received treatment with anti-PD-1 therapy, resulting in complete tumor regression and an expansion of the repertoire of neoantigen-specific T cells. These outcomes provide a compelling rationale for further development of this approach, either as a standalone therapy or in combination with checkpoint blockade or other immunotherapies [28]. In another study, researchers designed an mRNA vaccine targeting the CA-125 neoantigen for breast and ovarian cancer. The vaccine was tailored to cytotoxic CD8+ T cell epitopes based on somatic-mutation-driven neoantigens of CA-125 in breast or ovarian cancer. The researchers constructed a self-adjuvant mRNA vaccine that included CD40L- and MHC-I-targeting domains to enhance the cross-presentation of neoepitopes by dendritic cells [29].

### 3.3. Combating Resistance

Over time, tumors can develop resistance to ICIs. Neoantigen vaccines have the potential to diversify the immune response, thereby reducing the likelihood of resistance development. Acquired resistance to ICIs can arise through two primary mechanisms. (1) Impaired antigen presentation: this key resistance mechanism involves defects in the presentation of antigens by cancer cells. This hampers the immune system’s ability to identify and target these cells effectively. (2) Disrupted IFN-γ signaling pathway: Another critical resistance mechanism involves disruptions in the IFN-γ signaling pathway. This pathway plays a pivotal role in the immune response against cancer, and alterations in it can make tumors less responsive to immunotherapy [30,31]. To address these challenges, researchers are exploring the potential of neoantigen-based vaccination as a strategy to enhance the tumor immune microenvironment (TIME) and overcome resistance to ICIs. In a study involving six melanoma patients, notable results were obtained using the enzyme-linked immune absorbent spot (ELISPOT) assay. Neoantigen vaccination led to substantial ex vivo IFN-γ responses against a pool of neoepitopes. Importantly, this vaccination approach elicited polyfunctional de novo responses from both CD4+ and CD8+ T cells against neoepitope pools. These T cells demonstrated the ability to distinguish between mutated antigens and their corresponding wild-type epitopes, highlighting the specificity and efficacy of neoantigen-based vaccination [28]. In a separate study, researchers utilized functionally defined neoantigens (NeoAg) as targets for endogenous CD4+ and CD8+ T cells. Vaccines containing NeoAg recognized by both T cell subsets proved effective in overcoming resistance to immune checkpoint blockade (ICB). Particularly noteworthy was their ability to eradicate large established tumors, even those containing a subset of PD-L1+ tumor-initiating cancer stem cells (tCSCs), which are typically highly resistant to immune-based therapies [32]. These findings underscore the potential of neoantigen-based vaccination as a strategy to conquer acquired resistance to ICIs. This approach enhances cancer cell recognition, stimulates a potent anti-tumor T cell response, and effectively targets tumor-initiating cells, offering promise for advancing cancer immunotherapy outcomes.

Thus, the combination of neoantigen mRNA vaccines and ICI therapy represents a promising approach in the realm of cancer immunotherapy. This combination capitalizes on the personalized nature of neoantigen vaccines and the immune-boosting properties of ICIs to create a synergistic effect, potentially enhancing the immune system’s ability to target and eradicate cancer cells.

## 4. Current Research Stage of Applying Neoantigen mRNA Vaccine in HCC Treatment

### 4.1. Clinical Evidence of mRNA Vaccines Combined with ICIs in HCC Treatment

Cancer vaccines and various immunotherapies offer encouraging alternative approaches for the treatment of malignant tumors. Cancer vaccines can be engineered to focus on tumor-associated antigens, which are predominantly expressed in cancer cells, like growth-related factors, or antigens unique to malignant cells due to somatic mutations. These newly formed antigens, known as neoantigens, or the specific components within them, have been utilized as targets in human mRNA vaccine development. The majority of cancer vaccines are designed for therapeutic purposes rather than prevention and aim to activate a cell-mediated immune response. Numerous preclinical and clinical investigations have illustrated the feasibility of mRNA vaccines against cancer. Several categories of mRNA cancer vaccines are in various stages of development, each employing distinct approaches to target cancer cells. 

**Figure 2:**
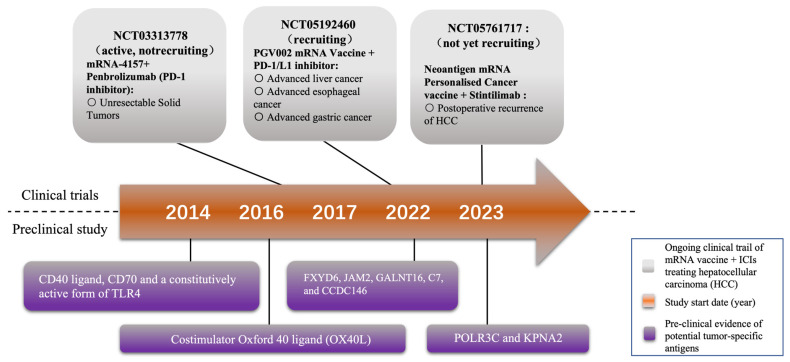
Milestones of selected mRNA vaccine research and development. At present, two mRNA vaccines are being tested in clinical trials for treating HCC combined with immune checkpoint inhibitors (ICIs). Some potential tumor-specific antigens for novel mRNA vaccine have also been reported by several preclinical studies.

Trial NCT05192460 is currently evaluating the safety and tolerance of the neoantigen tumor vaccine (PGV002 mRNA Vaccine). This study involves patients with advanced liver cancer, esophageal cancer, and gastric cancer. In detail, it is an investigator-initiated, single-center, open-label, single-arm exploratory study consisting of both a dose escalation phase and a dose expansion phase. In the dose escalation phase, patients receive the neoantigen tumor vaccine alone. Based on safety and efficacy data from this phase, the dose expansion phase is conducted at the intended clinical dose determined by the investigator. During this phase, the treatment involves a combination of the neoantigen tumor vaccine and PD-1/L1 inhibitors. The aim is to further evaluate the efficacy and safety profile of the neoantigen tumor vaccine at a specific dose when used in combination with PD-1/L1 inhibitors [33] (Figure 2) (Table 1).

In addition, trial NCT05761717, as an open, one-arm study, is focused on assessing the safety and effectiveness of an mRNA personalized tumor vaccine containing neonatal antigens when combined with Sintilimab injection. The primary goal is to prevent postoperative recurrence of HCC in the adjuvant setting. Specifically, this study follows a 3 + 3 dose escalation design. Participants in this trial will undergo a regimen that includes a total of six cycles of personalized cancer vaccine administered every 21 days. The neoantigen mRNA personalized cancer vaccine, in conjunction with Sintilimab injection, will be administered subcutaneously to the participants as part of this research effort (Figure 2) (Table 1).

Moreover, mRNA vaccine monotherapy is also being trialed in other studies. For instance, trial NCT05738447 represents a phase I study of an mRNA vaccine tailored for patients diagnosed with advanced HCC who are positive for the hepatitis B virus. This study identifies anti-HBV as a potential target for HCC. Another significant trial, NCT03480152, is a phase I/II clinical study that is currently ongoing. Its purpose is to assess the safety and immunogenicity of a multi-epitope mRNA vaccine designed for individuals with HCC and metastatic liver tumors [17]. In this study, researchers observed mild grade 1 and 2 toxicities, which promptly resolved, and importantly, no grade 3 or severe adverse events (SAEs) were reported. Additionally, the vaccine elicited both CD8 and CD4+ neoantigen-specific T cell responses. Notably, among the potential neoantigens tested, approximately 15.7% induced specific T cell immunity. Within this group, 59% of the neoantigens were recognized as CD4 epitopes, while the remaining 41% were identified as CD8 epitopes. These findings regarding the vaccine’s immunogenicity suggest the potential for treating patients with hepatocellular carcinoma (HCC) through combinations of the vaccine and other immune modulators, such as checkpoint inhibitors [17] (Table 1).

### 4.2. Potential Targets for Novel mRNA Vaccines 

The process of developing a personalized neoantigen vaccine entails the identification and confirmation of patient-specific somatic mutations that result in immunogenic non-synonymous changes within the tumor. These vaccines are customized based on the unique genetic makeup of a patient’s tumor. Researchers can create a vaccine that encodes the neoantigens produced by the identified tumor-specific mutations by sequencing the DNA of the tumor. The goal of this approach is to activate a focused immune response against cancer cells while leaving healthy cells unaffected. However, selecting the appropriate neoantigens is a challenging task that involves sequencing the patient’s tumor genome, identifying mutations, and predicting which ones will give rise to high-affinity neoantigenic peptides bound to the major histocompatibility complex (MHC).

**Figure 3:**
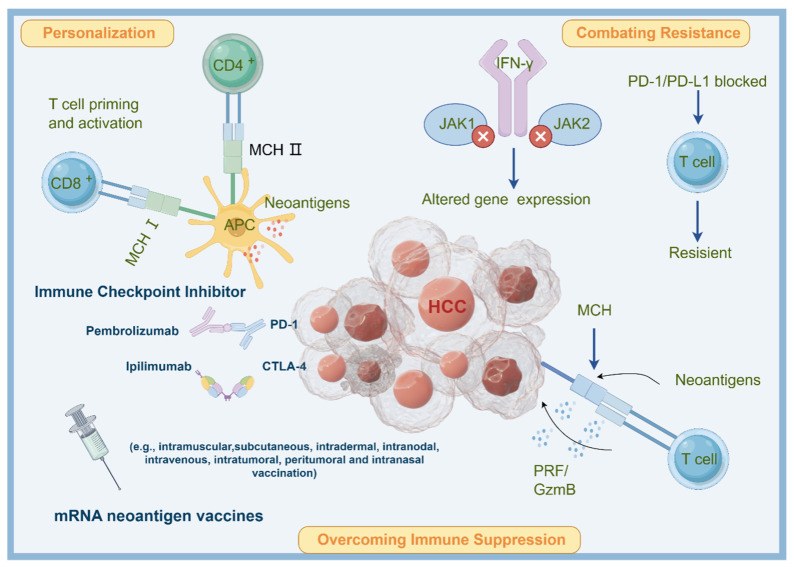
Potential mechanisms of applying neoantigen mRNA vaccine in HCC treatment. Neoantigens are unique mutations in cancer cells that can be targeted by personalized cancer vaccines. Neoantigen mRNA vaccines introduce synthetic mRNA encoding patient-specific neoantigens, which are translated into proteins by APCs. This activates T cells to eliminate cancer cells. Combining neoantigen vaccines with ICIs overcomes immune suppression, personalizes treatment, and combats resistance to ICIs. The combination enhances the immune response and improves outcomes in cancer immunotherapy.

Certain markers, specifically tumor-specific antigens (TSAs) and neoantigens, have emerged as promising candidates for the development of cancer vaccines [38]. TSAs are unique antigens exclusively present on the surface of cancer cells, setting them apart from healthy cells [39]. This distinctiveness allows the immune system to identify them as foreign or abnormal, prompting a robust immune response against the cancerous cells. TSAs can originate from various sources, including mutated proteins, known as neoantigens, or proteins that are overexpressed [40]. The presence of TSAs triggers the activation and proliferation of tumor-specific T cells, which hold the ability to directly target and eliminate cancer cells. Immune checkpoint inhibitors (ICIs) play a crucial role in enhancing this process by preventing T cell exhaustion and enabling their sustained activity. Notably, recent research has identified several novel TSAs in hepatocellular carcinoma (HCC) as potential therapeutic targets, including FXYD6, JAM2, GALNT16, C7, and CCDC146 [41]. Neoantigens, on the other hand, are protein fragments that result from somatic mutations occurring within cancer cells. These mutations lead to the formation of unique neoantigens that are specific to an individual’s cancer and are not subject to the immune system’s central tolerance mechanisms, which prevent the targeting of self-antigens [42]. Consequently, neoantigens represent ideal targets for immune responses. Given that ICIs are designed to block immune checkpoints that suppress the immune response, combining ICIs with neoantigens activates neoantigen-specific T cells, enabling them to effectively target cancer cells. Additionally, these T cells generated by ICIs can develop into memory T cells, providing long-lasting immunity against cancer cells. Furthermore, a higher number of neoantigens is often associated with a greater tumor mutational burden (TMB), enhancing the efficacy of ICI therapy (Figure 3).

Recent studies have identified novel HCC neoantigens and demonstrated the effectiveness of neoantigen-specific T cells against them through peptide-HLA (human leukocyte antigen) tetramer T-cell responses and in vitro ELISA assays. These findings confirmed the potent killing capabilities of HCC neoantigen-specific T cells in vitro [43]. Moreover, a novel neoantigen vaccine has shown promise in increasing the response rate of existing HCC immunotherapies [44]. This vaccine stimulates the production of polyfunctional T cells that specifically recognize mutated sequences and neoantigen-expressing cells, thus enhancing the immune response. Coimmunization experiments combining CD8+ and CD4+ neoantigen epitopes have also been successful in triggering robust CD8+ T cell responses. In addition, in vitro experiments have demonstrated the induction of immune responses against neoantigens in human HCC cells [44]. Furthermore, a recent clinical study applied neoantigen-reactive T cells combined with tomotherapy to treat a patient with advanced HCC (NCT03199807), resulting in a long-term progress-free survival, by enhancing the amount and anticancer effect of lymphocytes [45]. 

To further optimize the effectiveness of neoantigen vaccines, researchers have developed innovative technologies. For example, the low-toxicity cholesterol-modified antimicrobial peptide DP7-C serves a dual function as both a carrier and an immune adjuvant, improving the efficacy of dendritic cell (DC)-based vaccines. DP7-C has shown promising anti-tumor effects in mouse models. Notably, after DP7-C stimulation, the antigen uptake efficiency of monocyte-derived DCs (MoDCs) in patients with advanced lung cancer significantly increased, along with enhanced antigen presentation efficiency and the proportion of mature MoDCs. These advancements hold great potential for advancing cancer immunotherapy [46]. 

Additionally, a study has categorized immunological subtypes to identify groups that might benefit from cancer vaccination. The study suggests that patients with the IS1 immunological subtype may be appropriate candidates for vaccination. These findings suggest that immunotyping could potentially offer greater effectiveness in predicting the prognosis of hepatocellular carcinoma (HCC) patients compared to traditional tumor markers like Alpha-fetoprotein (AFP) and Glypican-3 (GPC3), as well as conventional staging methods [41].

Another study produced a map of genes that are expressed differently and mutated in HCC. This study also identified new antigens that are relevant to prognosis, including POLR3C and KPNA2. These antigens are considered promising candidates for mRNA vaccines, as their increased expression is linked to a poorer prognosis and high levels of B cells, macrophages, and dendritic cells. These observations indicate the significant involvement of these neoantigens in the progression and development of HCC. Patients who are categorized as having high immune infiltration in the IC3 group may benefit from mRNA vaccines. In both the TCGA and ICGC groups, the IC3 cluster had higher expression of ICD regulators, signifying a more robust immune response to mRNA vaccines. These findings form the basis for the advancement of anti-HCC mRNA vaccines, as well as for predicting prognosis and selecting suitable patients for vaccination [47]. 

Furthermore, a novel stable mRNA vaccine that can encode costimulator Oxford 40 ligand (OX40L) was synthesized and optimized. Based on the fact that OX40L is expressed on the cell surface and plays a role in co-stimulating T cells, the intratumoral injection of lipid nanoparticles (LNPs) containing OX40L mRNA has been shown to notably reduce tumor growth and enhance the survival of mice afflicted with HCC. In addition, this vaccine has displayed the ability to increase the level of CD4+ and CD8+ T cells in mice with HCC, suggesting a novel therapeutic approach for HCC immunotherapy through the utilization of mRNA vaccines [47].

A recent study has also reported the effectiveness of a TriMix mRNA, which encodes the CD40 ligand, CD70, and a constitutively active form of TLR4, in activating dendritic cells (DCs). This activation, in turn, creates a T-cell-attracting and stimulating environment. Additionally, when tumor antigen mRNA and TriMix are administered together in mice with HCC, it leads to the recruitment of CD4+ and CD8+ T cells that specifically target the antigen. Comparatively, delivering antigen mRNA alone, the concurrent administration of TriMix and antigen mRNA significantly increases the induction of antigen-specific T cells [48,49,50] (Figure 2).

Combining ICIs with therapies that promote the presentation of TSAs and neoantigens can enhance the immune system’s ability to recognize and target cancer cells specifically. These strategies hold promise in improving the therapeutic outcomes of immune checkpoint inhibitors and expanding their effectiveness to a broader range of cancer patients. Nevertheless, the identification of neoantigens in hepatocellular carcinoma (HCC) still presents a challenge. But with the emergence of next-generation sequencing technologies and advanced algorithms, the process of selecting neoantigens for personalized mRNA vaccines has become more attainable and feasible [51].

### 4.3. Novel Delivery for mRNA Vaccines 

Nanoparticles play a crucial role in the development of mRNA vaccines for cancer treatment. They serve as essential carriers for mRNA within vaccines. Given the rapid degradation of mRNA within the body, these nanoparticles are vital in shielding the mRNA, thereby ensuring its effective delivery to specific cells [52]. Moreover, the ability of nanoparticles to be precisely tailored allows them to home in on distinct cancer cell types, thereby boosting vaccine effectiveness. Alterations in their surface characteristics or the addition of unique ligands enable them to bind selectively to cancer-cell-specific receptors [53]. In addition, the role of nanoparticles in augmenting immune reactions is significant. By fine-tuning their size and surface traits, nanoparticles can be made more amenable to absorption by dendritic cells, which are instrumental in triggering an immune response [54]. Nanoparticles also facilitate a measured release of mRNA, which is crucial for a persistent and effective immune response. This regulation is key in maintaining an appropriate antigen level for optimal stimulation of the immune system [55]. Furthermore, the focused delivery of the vaccine to specific cells via nanoparticles minimizes the likelihood of side effects. This precision means that lower doses of the vaccine are required for therapeutic impact, further reducing the risk of adverse effects. Certain nanoparticles also possess adjuvant qualities, meaning they can independently bolster the immune system’s response to the vaccine [56]. This dual functionality not only enhances effectiveness but also streamlines the vaccine formulation process [57].

In numerous instances, lipid nanoparticles or protein-based polyplexes have been employed to amplify the therapeutic potential of mRNA vaccines. These carriers aid in expressing tumor antigens, which activate cytotoxic immunity, and they also upregulate genes associated with cancer. This dual action results in tumor cell death and the suppression of tumor growth. 

In addition, exploration of novel NP-assisted mRNA delivery systems is ongoing. For instance, recent evidence has revealed a new nanoparticle that targets CXCR4, which is expressed on liver cancer cells. Such a material can selectively deliver mRNA for the p53 gene to liver cancer cells. Therefore, this nanocarrier has been used to deliver p53 mRNA in combination with an anti-PD-1 monoclonal antibody, which restores p53 expression, induces reprogramming of the tumor microenvironment, and successfully inhibits the development of HCC in vivo. It has prolonged survival and reduced liver ascites and metastases in mice with liver cancer. Moreover, the anti-tumor impact of this combined treatment surpasses the effects of either the anti-PD-1 monoclonal antibody or therapeutic P53 expression alone [58].

### 4.4. mRNA Vaccine for HCC Preventive Therapies 

Due to the low survival rates among individuals with advanced HCC, as well as the rising number of patients with chronic liver disease and the high incidence of liver tumors, it is crucial to develop effective prevention strategies. In this context, mRNA vaccines have also been considered as a potential preventive approach for liver cirrhosis and HCC. 

For instance, evidence has shown that when specific double-stranded RNA (dsRNA), polyinosinic-polycytidylic acid (pIC), was injected into mice, a model of liver cancer at the pre-cancerous stage, there was growth inhibition of liver cancer cells in the mice. By injecting PIC, the preventive effect on liver cancer was more significant than the therapeutic effect. Moreover, PIC injection has been found to reduce the expression of certain biomarkers (such as CD44v6, Sox9, A6, epithelial cell adhesion molecules (EpCAM)) that are associated with the inhibition of liver tumorigenesis. These findings create a foundation for the utilization of non-specific immunostimulatory agents, like synthetic dsRNA, which might potentially help deter the onset of HCC in individuals with high-risk fatty liver conditions [59]. 

Although mRNA has the potential to elicit a robust cellular immune response, there have not been any reported successful therapeutic vaccines for HCC thus far. Similarly, a strong cellular immune response would be advantageous in addressing patients with chronic hepatitis B, but no mRNA-based therapeutic vaccine for this purpose has been documented. Undoubtedly, the most significant challenge in treating liver cancer and chronic hepatitis B is the creation of an effective immune response within the liver’s distinct and immune-tolerant environment. It is worth noting that HCC is a complex disease with a diverse set of underlying causes, including hepatitis B and C infection, alcohol consumption, and obesity. Successful vaccine development for HCC may require a multifaceted approach that addresses these underlying causes as well as the unique characteristics of HCC tumors. While the translation of these therapeutic effects to human clinical trials is a matter that needs to be addressed, mRNA vaccines offer a new and promising approach for the treatment and prevention of HCC.

### 4.5. Potential Adverse Drug Reactions of mRNA Vaccines

As discussed above, mRNA vaccines apparently present as a hopeful strategy in treating liver cancer, yet their potential side effects warrant careful consideration. For instance, mRNA vaccines activate the immune system, which can sometimes lead to an excessive immune reaction [60]. This hyperactivity might result in inflammation or autoimmune responses, adversely affecting liver function, especially in those with existing liver ailments. In addition, individuals with pre-existing liver conditions may face a heightened risk of liver toxicity [61]. The liver’s crucial role in metabolizing and eliminating substances means that an intensified immune response or additional strain could aggravate liver injuries or disrupt liver functionality. Moreover, similar to other vaccines, mRNA vaccines carry a risk of allergic reactions that can vary from mild to intense [62]. Ingredients in the vaccine, such as lipid nanoparticles, could be the triggers for these allergic responses [63].

Typical reactions at the injection area, including soreness, redness, or swelling, are possible. These symptoms are usually short-lived but could be more intense in some cases [64]. Additionally, systemic symptoms like fever, tiredness, headaches, and muscle aches are often reported. For liver cancer patients, these general reactions could further complicate their overall health condition [65]. There is a possibility that mRNA vaccines could interact with other liver cancer therapies, such as chemotherapy or targeted treatments, potentially impacting the treatment’s efficacy or intensifying side effects [66].

To address those potential adverse drug reactions in liver cancer patients, several promising strategies may be considered in future studies. For instance, developing personalized vaccine protocols based on individual health profiles and pre-existing conditions [67]; administering medications to mitigate allergic reactions or immune hyperactivity before vaccine administration [68]; and researching and developing vaccine formulations that are less likely to cause intense immune reactions or liver toxicity [69]. In addition, providing supportive care for managing systemic symptoms like fever, tiredness, and muscle aches to minimize their impact on overall health may also be feasible [70]. Apparently, continuous research is strongly required to assess the safety and effectiveness of mRNA vaccines in liver cancer therapy. Medical decisions should also be individualized, taking into account each patient’s general health, liver function, and the specific nature of their cancer.

## 5. Conclusions

As research progresses, it is likely that new types of mRNA cancer vaccines will emerge with the goal of maximizing their therapeutic potential and overcoming the challenges associated with cancer immunotherapy. While the identification of immunogenic TAAs/TSAs, as well as overcoming the inhibitory tumor microenvironment, continues to pose significant challenges for mRNA vaccines, recent advancements in the discovery and identification of neoantigens have provided more possibilities for personalized vaccine treatments.

mRNA-encoded neoantigens have emerged as leaders in the realm of personalized cancer vaccines. Multiple clinical studies conducted using mRNA LNPs have already yielded promising outcomes, demonstrating anti-tumor immune responses, and have been applied in various clinical trials to address various solid tumors. This marks the onset of a new era for therapeutic cancer vaccines. To enhance the efficacy of mRNA anticancer vaccines, several ongoing clinical trials are assessing their combination with cytokine therapies or checkpoint inhibitor therapies. 

Furthermore, future research endeavors should focus on various aspects, including understanding and harnessing the inherent innate immune responses triggered by mRNA, enhancing the efficiency of antigen expression and presentation through advanced and well-tolerated delivery systems, and modifying mRNA structures to achieve extended and controlled duration of expression.

The integration of mRNA vaccines into liver cancer treatment presents significant potential, yet it is not without its challenges within the pharmaceutical industry, which need careful attention. The mass production of mRNA vaccines presents a complex challenge, necessitating the development of methods that are both efficient and cost-effective to ensure widespread accessibility. Furthermore, these vaccines typically demand specialized, ultra-low temperature storage conditions, creating substantial logistical hurdles, particularly in areas with less developed infrastructure. Navigating the regulatory landscape for the approval of mRNA vaccines in cancer treatment is another complex and time-consuming process. Ensuring safety and efficacy while adhering to these regulatory requirements is vital for industry progress. Additionally, the inherent costs associated with the development and production of these vaccines pose a significant obstacle in making them affordable and accessible to a wider population. Given the relatively recent emergence of mRNA technology in cancer treatment, there is a crucial need for long-term safety and efficacy data, necessitating extensive and resource-heavy research and clinical trials. The diverse genetic and molecular profiles of liver cancer also demand personalized treatment approaches. Crafting mRNA vaccines to meet individual patient needs is a complex and costly endeavor. There is also the concern of the immune system’s potential overreaction or tolerance to these vaccines, which could impact their effectiveness in certain patients. Understanding and addressing these immune responses is key to the success of mRNA vaccines in treating liver cancer. Overcoming these challenges is critical for the effective implementation of mRNA vaccines in liver cancer therapy. It requires a concerted effort from not just the pharmaceutical industry, but also regulators and the wider scientific community.

To sum up, mRNA is a powerful and versatile cancer vaccine platform. Its successful development towards clinical translation will remarkably strengthen our ability to combat cancers. Fortunately, there has been notable progress in the field of using neoantigen mRNA vaccines in combination with ICIs for the treatment of HCC. Combined with the results achieved in the KEYNOTE-942 trial, this synergistic combination may also represent a novel breakthrough in the treatment of HCC. However, very limited evidence has been reported in this field at present. Therefore, the objective of this commentary is to inspire further exploration of the potential application of this therapeutic strategy for HCC. 

## Figures and Tables

**Table 1:** Basic information of selected clinical trials.

Year	NCT Number	Study Status	Phase	Cancer Type	Number of Patients	Ref.
**2022**	NCT05192460	Recruiting	N/A	Gastric Cancer Esophageal Cancer Liver Cancer	30	[34]
**2023**	NCT05761717	Not yet recruiting	N/A	Postoperative Hepatocellular Carcinoma	67	[35]
**2023**	NCT05738447	Recruiting	I	Liver Cancer Hepatocellular Carcinoma	9	[36]
**2018**	NCT03480152	Terminated (slow accrual)	I/II	Melanoma Colon Cancer Gastrointestinal Cancer Genitourinary Cancer Hepatocellular Cancer	5	[37]

## Data Availability

Data is contained within the article.
